# A Mind Free to Wander: Neural and Computational Constraints on Spontaneous Thought

**DOI:** 10.3389/fpsyg.2019.00039

**Published:** 2019-01-23

**Authors:** Elisa Ciaramelli, Alessandro Treves

**Affiliations:** ^1^Dipartimento di Psicologia, Università di Bologna, Bologna, Italy; ^2^SISSA - Cognitive Neuroscience, Trieste, Italy

**Keywords:** mind-wandering, attention, episodic memory retrieval, ventromedial prefrontal cortex, hippocampus, neuropsychology, computational modeling

One prominent feature of human conscious experience is mind-wandering, the automatic drift of attention away from an ongoing task toward thoughts often completely unrelated to the task at hand (e.g., thinking about running while reading a manuscript; Antrobus et al., 1966; Christoff et al., 2016). Humans spend about 25–50% of their daily lives mind-wandering (Killingsworth and Gilbert, 2010), with obvious disadvantages for performance on ongoing tasks (Smallwood and Schooler, 2015). We do not mind-wander so frequently because it is fun. In fact, mind-wandering most often causes bad mood, regardless of whether the content of off-task thoughts was negative or positive (Killingsworth and Gilbert, 2010). Rather, mind-wandering may be adaptive. For example, after an incubation period filled with a trivial task promoting mind-wandering, individuals were better at conceiving unusual uses of common objects, as if mind-wandering favored the unconscious, unconstrained interaction of multiple, distant concepts typical of creative thinking (Baird et al., 2012). This advantage was not observed if during the incubation period participants rested, possibly because rest increases the tendency to think (consciously) about the terms of the problem, constraining excessively the content of thought.

Mind-wandering may have an internal origin (be self-initiated), but it can also be triggered by external cues (e.g., reading the word “experiment” leads to think to try and become a runner; e.g., McVay and Kane, 2013; Maillet et al., 2017; Vannucci et al., 2017). Once initiated, either because internally or externally cued, mind-wandering tends to unfold in a rather unconstrained fashion, with a peculiar phenomenology consisting—to say it with James (1890)—of birds' perchings and flights, with perchings being the discrete contents of thought on which memory retrieval converges (and attention focuses), and flights being the transitions of attention from one content to the next. How do we mind-wander? Which brain regions and dynamics govern the triggering of mind-wandering and its trajectories in the space of thoughts?

In the last decade, there has been an increasing interest in the cognitive and neural mechanisms of mind-wandering and other forms of spontaneous cognition, after the field had been dominated by the study of goal-directed cognition. Functional neuroimaging (fMRI) evidence indicates that mind-wandering is associated with activity in the “default network,” a set of interconnected brain regions, including the medial temporal lobes (MTLs), ventromedial prefrontal cortex (vmPFC), posterior cingulate cortex, and the angular gyrus, whose activity is enhanced during relatively passive states and internally focused thought (Buckner et al., 2008; Christoff et al., 2009; Smallwood et al., 2012; Fox et al., 2015). According to one prominent view, activity in the default network is related to the production of the mental contents populating mind-wandering, with separate subsystems mediating the memory-based construction of mental events and their self-relevant connotation (Andrews-Hanna et al., 2010). An alternative view, sprung from the observation that the default network is active during the unfocused monitoring of external events (Gilbert et al., 2006a), is that activity in this network does not necessarily reflect mind-wandering, but, more in general, the capture of attention by salient task-unrelated stimuli, which also includes external distractions (e.g., noises). This latter view relates to the “gateway hypothesis” of medial prefrontal cortex as implicated in orchestrating the allocation of attention between internal and external events, and its monitoring/awareness (Gilbert et al., 2006b). There is initial fMRI evidence, however, that medial prefrontal cortex is generally more engaged by mind-wandering than by external distractions, though different subregions of medial prefrontal cortex respond preferentially to different forms of distraction (Stawarczyk et al., 2011).

One important question is whether activity in key nodes of the brain default network is necessary for mind-wandering. Lesion studies can relate brain activity causally with behavior, and constrain the interpretation of the function of targeted brain regions in a way that is not possible with neuroimaging data alone. The results from two neuropsychological studies of mind-wandering in patients with bilateral damage in vmPFC vs. the hippocampus are initial evidence that these two regions play necessary but distinct roles in mind-wandering. Bertossi and Ciaramelli (2016) had vmPFC patients and brain-damaged and healthy controls perform various tasks varying in difficulty, hence conduciveness to mind-wandering. Across tasks, participants were occasionally probed to report whether their thoughts had been fully on-task or, to some extent, off-task, and about the contents of off-task thoughts. They found that vmPFC patients showed a reduced frequency of mind-wandering, and, when they did mind-wander, their thoughts were mostly about the present, never about the future. Interestingly, vmPFC damage did not change the frequency with which participants claimed they were unaware of the content of their off-task thoughts, suggesting it caused impaired construction, not meta-awareness, of mind-wandering contents (see also Bertossi et al., 2017). We are currently collecting indirect (physiological) indices of mind-wandering in vmPFC patients to clarify whether lack of meta-awareness contributed to reduced mind-wandering. McCormick et al. (2018b), on the other hand, examined mind-wandering in patients with hippocampal damage probing the contents of their thoughts over a 2-day period. They found that hippocampal patients reported mind-wandering as frequently as controls. However, off-task thoughts were context-rich (episodic) in healthy controls, but semanticized and mainly present-oriented in hippocampal patients. Although the designs of the two studies differs, the results suggest that vmPFC patients are impaired at decoupling from the external environment and initiating mind-wandering, whereas hippocampal patients do engage in mind-wandering, but have it devoid of episodic content. We tentatively proposed, therefore, that during mind-wandering (as well as voluntary event construction), vmPFC initiates the construction of events alternative to direct (perceptual) experience, by coordinating the activation of relevant schemata (e.g., the park where I run; Gilboa and Marlatte, 2017), which the hippocampus uses to build a rudimentary sketch of the event. vmPFC would then help fill the mental event by engaging in iterative retrieval and integration of schema-congruent elements via feedback loops with the hippocampus and neocortex (e.g., what typically happens when I run; see also Benoit et al., 2014; Moscovitch et al., 2016; McCormick et al., 2018a).

Yet, vmPFC patients may not be pervasively unable to mind-wander. Ciaramelli and Ghetti (2007) observed that in recognition memory tasks vmPFC patients tend to falsely recognize test (distractor) items because they make vivid but task-irrelevant associations during retrieval (e.g., I remember the word CUP because I bought a red cup in London). These vivid associations may in fact be instances of externally-triggered mind-wandering, and evidence that this form of mind-wandering is still possible following vmPFC damage, consistent with fMRI evidence (Stawarczyk et al., 2011). Similarly, in explicit memory tasks, vmPFC patients may fail to retrieve any memory, and then start confabulating floridly if probed (Moscovitch and Melo, 1997). Thus, vmPFC patients' mind-wandering and event construction seem to depend critically on the presence of external cues, whose availability determines striking qualitative changes in patients' behavior.

Another dissociation between vmPFC and hippocampal patients is worth mentioning here. Kurczek et al. (2015) investigated episodic remembering and future thinking in vmPFC vs. hippocampal patients by having them first produce past/future events and then select one moment from the event and describe it in detail. Individuals with MTLs damage were unable to describe such moments in detail, but vmPFC patients could. In another study, however, Bertossi et al. (2016) required vmPFC patients to construct entire past and future events, and found a striking impairment. These findings suggest that hippocampal patients are impaired in conjuring up even single scenes/moments from an event, while vmPFC patients may be impaired in constructing extended events (McCormick et al., 2018a). This dissociation, too, points to differences in the role of vmPFC and the hippocampus in the dynamic construction of the flow of thoughts, with the hippocampus contributing the discrete contents of events (the perchings) and vmPFC prescribing the appropriate transition between moments of the events (the flights). What is unclear is what allows vmPFC and the hippocampus to contribute to mind-wander differentially, whether their specialized internal organization or merely their sitting at the appropriate confluence of the relevant information streams. Characterizing the cortical dynamics of mind-wandering may be helped by combining behavioral studies in brain-lesioned patients with mathematically defined network models incorporating core principles of cortical organization. Comparing the functional contribution of vmPFC and the hippocampus acquires extra significance, given the contrast between the neocortical architecture of the former vs. the peculiar internal organization of the latter, centered on the unique characteristics of the dentate gyrus (Treves et al., 2008) and on the CA3-CA1 differentiation (Treves, 2004).

At a very general level, streams of thought may be conceived as trajectories among declarative memories. Mathematical models of memory storage and retrieval in the hippocampus, pioneered by Marr (1971) and later empowered by the analysis of the Hopfield model (Amit et al., 1987), conceive episodic memories as attractor states in the CA1 and CA3 regions of the hippocampus. While the intrinsic CA3 connectivity would enable the cued retrieval of temporally-defined scenes of arbitrary content, CA1 may allow for their limited temporal association, e.g., the concatenation of scenes within an episode (Kesner et al., 2002; Treves, 2004). Local recurrent connectivity within cortical regions is thought to endow them, too, with attractor states, with contents specific to each region. The collection of local attractor networks can engage in “latching” dynamics, when in response to a cue the whole neocortex does not just settle into a single attractor (whether instantaneous like a snapshot or somewhat extended in time), but instead continues to hop from one attractor to the next (Treves, 2005). Mathematically defined “Potts” networks have been shown to undergo, depending on their parameters (e.g., number of units, number of states they are endowed with; Kang et al., 2017), phase transitions—abrupt changes in their dynamics—passing from a “no latching” region to a “finite latching” region, to an “infinite latching” region, in which latching dynamics go on spontaneously and indefinitely (Russo and Treves, 2012; Naim et al., 2018). Importantly, current work is analyzing how such spontaneous hopping may be supplemented by schemata stored in parts of the extended network, e.g., vmPFC.

Mind-wandering (as well as the conflation of memories in confabulation) is reminiscent of a latching process in which some of the transitions appear random, others rather more guided by local schemata. We propose that vmPFC participates in the mechanics of neocortical latching, facilitating congruent consecutive retrieval of stored memories, while their content is boosted by the hippocampus. vmPFC-mediated transitions between contents of thought would occur through the instantiation of specific local schemata (see Gilboa and Marlatte, 2017, for a review). Mathematizing the psychological concept, a “schema” may be conceived as the association of attractor state *k* in local network *i* with the subsequent attractor state *l* in local network *j*, an association extracted over multiple similar occurrences (Gilboa and Marlatte, 2017). If $\sigma_{i}^{k}$ denotes the activation of attractor *k* in network *i*, the schema could be instantiated in a Potts network by adding to its “free-energy” function a term proportional to $\sigma_{i}^{k}$·$\sigma_{j}^{l}$, which would cause substantial interference among memories. The latching Potts network, however, naturally envisages additional ramping variables $\theta_{i}^{k}$, which parametrize how long a temporally extended attractor $\sigma_{i}^{k}$ has been activated. A free-energy term proportional to $\theta_{i}^{k}$·$\sigma_{j}^{l}$facilitates schema-guided transitions, in relation to the contents represented by local networks *i* and *j* (for example, in vmPFC, or in Broca's area), while the remaining content may be stationary, or undergo spontaneous transitions, or be guided by other schemata. On the other hand, a Potts model connected with a hippocampal model may utilize it as an “episodic content booster,” reinvigorating streams of thoughts in the cortex, and is expected to show saltatory characteristics, in that hippocampal output representations would be activated not too frequently relative to the sequence of neocortical states.

Despite many open issues requiring detailed model analysis, we expect it to support our view that the hippocampus fuels voluntary as well as spontaneous cognition with detail-rich scenes/snapshots, whereas vmPFC (among areas storing specific schemata) governs appropriate latching across memory attractors to form extended events. Our model and related predictions are portrayed in Figure 1. In healthy controls, attention shifts from an ongoing task inward, toward mentally constructed experiences. These flow, guided by relevant schemata and boosted by context-rich hippocampal memories. A lesion to the hippocampal component is expected to result in reduced episodic content boosts, with preserved schema-driven transitions: the flow of thoughts now “browses” on context-poor items/moments. Conversely, a lesion to vmPFC is expected to disarticulate mind-wandering, leaving it over-dependent on the hippocampal content booster: ephemeral, inconsequential mind-wandering is now triggered by the infrequent hippocampal output and poorly assisted by schema-guided construction processes.

**Figure 1:**
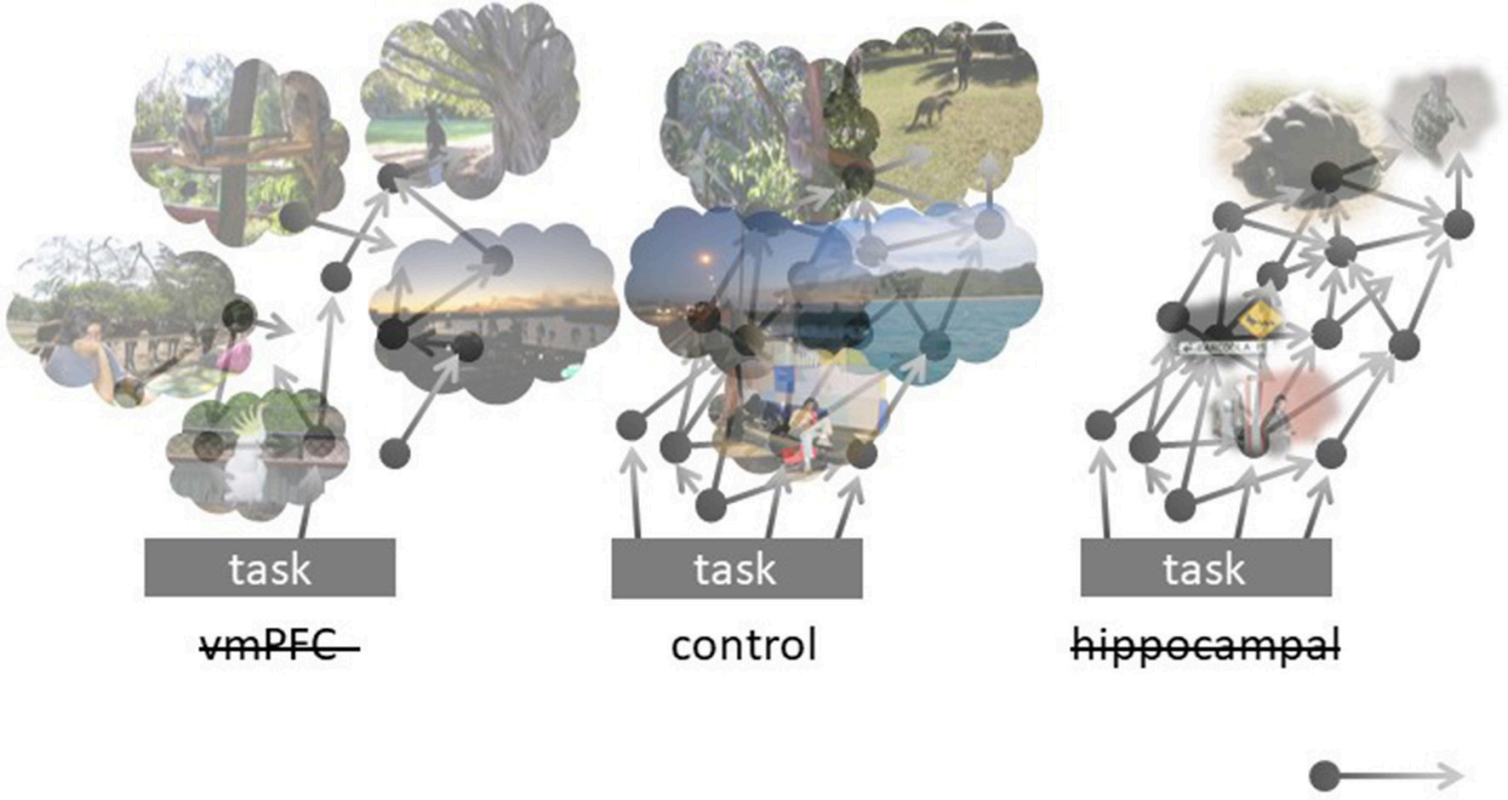
Schematic representation of the model implemented in the Potts network, in its control **(center)**, vmPFC-lesioned **(left)**, and hippocampally-lesioned **(right)** versions. The black circles represent schemata, some of which are stored as continuous attractors in vmPFC, that often lead (arrows) to the activation of other schemata, in multiple interacting sequences. Ongoing activity in the cortex sometimes elicits the activation of memories in the hippocampus (clouds), boosting its contents with episodic details. Mind-wandering can be initiated by schemata activated by aspects of the current task or endogenously, or by the direct activation of episodic memories, particularly in vmPFC patients, in which the chain of continuous attractors is weakened. Hippocampal patients, on the other hand, experience rarer and weaker episodic boosting of their mind-wandering chains.

Future neuropsychological studies and computational analyses will help test and refine the model and clarify the role of vmPFC vs. the hippocampus in the dynamics of mind-wandering. For example, if vmPFC is necessary to initiate and maintain mind-wandering endogenously, vmPFC lesions should lead to reduced mind-wandering when no cue is provided or no strong memory attractor is probed (due to weak schema-assisted latching), but preserve mind-wandering if externally cued, especially in response to highly imaginable words or strong words probing schemas (e.g., the self, one's own goals), which both act as strong retrieval cues (McVay and Kane, 2013; Vannucci et al., 2017). With hippocampal lesions, external cues are expected to be less beneficial. Another prediction pertains to the temporal trajectory and content of mind-wandering following an external trigger. In patients/networks with vmPFC lesions, mind-wandering is expected to be largely limited to short delays after the external cue (weak latching), and to often depart from the schema probed by the cue, to reflect diverse hippocampal output. Conversely, in patients/networks with hippocampal lesions, mind-wandering should be more long-lasting but more constrained in content to schema-instructed latching dynamics.

Observing a phase-dependent behavior as in a Potts network (endowed with a hippocampal content booster) would substantially improve our understanding of the role of vmPFC in the schema-driven temporal development of mind-wandering and constructed experience. An abnormal balance between spontaneous and schema-guided latching dynamics may account for the somewhat paradoxical behaviors of vmPFC patients, who range, depending on the experimental condition, from being unable to retrieve any memory or engage in mind-wandering to floridly confabulate and make off-task associations (Moscovitch and Melo, 1997; Ciaramelli and Ghetti, 2007; Bertossi and Ciaramelli, 2016). Understanding the model may also facilitate clinical applications. Previous attempts to reduce confabulation have reinforced retrieval goals (Ciaramelli, 2008) or muffled the cognitive resources available for task-irrelevant associations (Ciaramelli et al., 2009). In hindsight, we were acting on parameters of a Potts network—what we can now do explicitly, to study, and hopefully manage thought-flow impairments.

## Author Contributions

EC and AT conceived, wrote, and provided approval for publication of this work.

### Conflict of Interest Statement

The authors declare that the research was conducted in the absence of any commercial or financial relationships that could be construed as a potential conflict of interest.

## References

[B1] Amit D. J. Gutfreund H. Sompolinsky H. (1987). Statistical mechanics of neural networks near saturation. Ann. Phys. 173, 30–67. 10.1016/0003-4916(87)90092-3

[B2] Andrews-Hanna J. R. Reidler J. S. Sepulcre J. Poulin R. Buckner R. L. (2010). Functional-anatomic fractionation of the brain's default network. Neuron 65, 550–562. 10.1016/j.neuron.2010.02.005 20188659 PMC2848443

[B3] Antrobus J. S. Singer J. L. Greenberg S. (1966). Studies in the stream of consciousness: experimental enhancement and suppression of spontaneous cognitive processes. Percept. Mot. Skills 23, 399–417. 10.2466/pms.1966.23.2.399

[B4] Baird B. Smallwood J. Mrazek M. D. Kam J. W. Franklin M. S. Schooler J. W. (2012). Inspired by distraction: mind wandering facilitates creative incubation. Psych. Sci. 23, 1117–1122. 10.1177/0956797612446024 22941876

[B5] Benoit R. G. Szpunar K. K. Schacter D. L. (2014). Ventromedial prefrontal cortex supports affective future simulation by integrating distributed knowledge. Proc Natl. Acad. Sci. U.S.A. 111, 16550–16555. 10.1073/pnas.1419274111 25368170 PMC4246308

[B6] Bertossi E. Ciaramelli E. (2016). Ventromedial prefrontal damage reduces mind-wandering and biases its temporal focus. Soc. Cogn. Affect. Neurosci. 11, 1783–1791. 10.1093/scan/nsw099 27445210 PMC5091689

[B7] Bertossi E. Peccenini L. Solmi A. Avenanti A. Ciaramelli E. (2017). Transcranial direct current stimulation of the medial prefrontal cortex dampens mind-wandering in men. Sci. Rep. 7:16962. 10.1038/s41598-017-17267-4 29209069 PMC5717259

[B8] Bertossi E. Tesini C. Cappelli A. Ciaramelli E. (2016).Ventromedial prefrontal damage causes a pervasive impairment of episodic memory and future thinking. Neuropsychologia 81, 107–116. 10.1016/j.neuropsychologia.2015.12.015 26827916

[B9] Buckner R. L. Andrews-Hanna J. R. Schacter D. L. (2008). The brain's default network: anatomy, function, and relevance to disease. Ann. N. Y. Acad. Sci. 1124, 1–38. 10.1196/annals.1440.011 18400922

[B10] Christoff K. Gordon A. M. Smallwood J. Smith R. Schooler J. W. (2009). Experience sampling during fMRI reveals default network and executive system contributions to mind wandering. *Proc. Natl. Acad. Sci*. U.S.A. 106, 8719–8724. 10.1073/pnas.0900234106 PMC2689035 19433790

[B11] Christoff K. Irving Z. C. Fox K. C. Spreng R. N. Andrews-Hanna J. R. (2016). Mind-wandering as spontaneous thought: a dynamic framework. Nat. Rev. Neurosci. 17, 718–731. 10.1038/nrn.2016.113 27654862

[B12] Ciaramelli E. (2008). The role of ventromedial prefrontal cortex in navigation: a case of impaired wayfinding and rehabilitation. Neuropsychologia 46, 2099–2105. 10.1016/j.neuropsychologia.2007.11.029 18201735

[B13] Ciaramelli E. Ghetti S. (2007). What are confabulators' memories made of? A study of subjective and objective measures of recollection in confabulation. Neuropsychologia 45, 1489–1500. 10.1016/j.neuropsychologia.2006.11.007 17222872

[B14] Ciaramelli E. Ghetti S. Borsotti M. (2009). Divided attention during retrieval suppresses false recognition in confabulation. Cortex 45, 141–153. 10.1016/j.cortex.2007.10.006 19150516

[B15] Fox K. C. Spreng R. N. Ellamil M. Andrews-Hanna J. R. Christoff K. (2015). The wandering brain: meta-analysis of functional neuroimaging studies of mind-wandering and related spontaneous thought processes. Neuroimage 111, 611–621. 10.1016/j.neuroimage.2015.02.039 25725466

[B16] Gilbert S. J. Simons J. S. Frith C. D. Burgess P. W. (2006a). Performance-related activity in medial rostral prefrontal cortex (area 10) during low-demand tasks. J. Exp. Psychol. Hum. Percept. Perform. 32, 45–58. 10.1037/0096-1523.32.1.45 16478325

[B17] Gilbert S. J. Spengler S. Simons J. S. Steele J. D. Lawrie S. M. Frith C. D. (2006b). Functional specialization within rostral prefrontal cortex (area 10): a metaanalysis. J. Cogn. Neurosci. 18, 932–948. 10.1162/jocn.2006.18.6.932 16839301

[B18] Gilboa A. Marlatte H. (2017). Neurobiology of Schemas and Schema-mediated memory. Trends Cogn. Sci. 21, 618–631. 10.1016/j.tics.2017.04.013 28551107

[B19] James W. (1890). The Principles of Psychology. New York, NY: Henry Holt and Company.

[B20] Kang C. J. Naim M. Boboeva V. Treves A. (2017). Life on the edge: latching dynamics in a potts neural network. Entropy 19:468 10.3390/e19090468

[B21] Kesner R. P. Gilbert P. E. Lee I. (2002). Subregional analysis of hippocampal function in the rat, in Neuropsychologyof Memory. 3rd Edn, eds Squire L. R. Schacter D. L. (New York, NY: Guilford Press), 395–411.

[B22] Killingsworth M. A. Gilbert D. T. (2010). A wandering mind is an unhappy mind. Science 330:932 10.1126/science.1192439 21071660

[B23] Kurczek J. Wechsler E. Ahuja S. Jensen U. Cohen N. J. Tranel D.. (2015). Differential contributions of hippocampus and medial prefrontal cortex to self-projection and self-referential processing. Neuropsychologia 73, 116–126. 10.1016/j.neuropsychologia.2015.05.002 25959213 PMC4671497

[B24] Maillet D. Seli P. Schacter D. L. (2017). Mind-wandering and task stimuli: stimulus-dependent thoughts influence performance on memory tasks and are more often past- versus future-oriented. Conscious Cogn. 52, 55–67. 10.1016/j.concog.2017.04.014 28460272 PMC5494999

[B25] Marr D. (1971). Simple memory: a theory of archicortex'. Phil. Trans. B 370:1666. 10.1098/rstb.1971.0078 4399412

[B26] McCormick C. Ciaramelli E. De Luca F. Maguire E. A. (2018a). Comparing and contrasting the cognitive effects of hippocampal and ventromedial prefrontal cortex damage: a review of human lesion studies. Neuroscience 374, 295–318. 10.1016/j.neuroscience.2017.07.066 28827088 PMC6053620

[B27] McCormick C. Rosenthal C. R. Miller T. D. Maguire E. A. (2018b). Mindwandering in people with hippocampal damage. J. Neurosci. 38, 2745–2754. 10.1523/JNEUROSCI.1812-17.2018 29440532 PMC5851780

[B28] McVay M. Jc. Kane M. J. (2013). Dispatching the wandering mind? Toward a laboratory method for cuing “spontaneous” off-task thought. Front. Psychol. 4:570. 10.3389/fpsyg.2013.00570 24027542 PMC3760067

[B29] Moscovitch M. Cabeza R. Winocur G. Nadel L. (2016). Episodic memory and beyond: the hippocampus and neocortex in transformation. Annu. Rev. Psychol. 67, 105–134. 10.1146/annurev-psych-113011-143733 26726963 PMC5060006

[B30] Moscovitch M. Melo B. (1997). Strategic retrieval and the frontal lobes: evidence from confabulation and amnesia. Neuropsychologia 35, 1017–1034. 10.1016/S0028-3932(97)00028-6 9226662

[B31] Naim M. Boboeva V. Kang C. J. Treves A. (2018). Reducing a cortical network to a Potts model yields storage capacity estimates. J. Stat. Mech. 2018:043304 10.1088/1742-5468/aab683

[B32] Russo E. Treves A. (2012). Cortical free-association dynamics: distinct phases of a latching network. Phys. Rev. E 85:051920. 10.1103/PhysRevE.85.051920 23004800

[B33] Smallwood J. Brown K. Baird B. Schooler J. W. (2012). Cooperation between the default mode network and the frontal-parietal network in the production of an internal train of thought. Brain Res. 1428, 60–70. 10.1016/j.brainres.2011.03.072 21466793

[B34] Smallwood J. Schooler J. W. (2015). The science of mind wandering: empirically navigating the stream of consciousness. Annu.Rev.Psychol. 66, 487–518. 10.1146/annurev-psych-010814-015331 25293689

[B35] Stawarczyk D. Majerus S. Maquet P. D'Argembeau A. (2011). Neural correlates of ongoing conscious experience: both task-unrelatedness and stimulus-independence are related to default network activity. PLoS ONE 6:e16997. 10.1371/journal.pone.0016997 21347270 PMC3038939

[B36] Treves A. (2004). Computational constraints between retrieving the past and predicting the future, and the CA3-CA1 differentiation. Hippocampus 14, 539–556. 10.1002/hipo.10187 15301433

[B37] Treves A. (2005). Frontal latching networks: a possible neural basis for infinite recursion. Cogn. Neuropsychol. 22, 276–291. 10.1080/02643290442000329 21038250

[B38] Treves A. Tashiro A. Witter M. P. Moser E. I. (2008). What is the mammalian dentate gyrus good for? Neuroscience 154, 1155–1172. 10.1016/j.neuroscience.2008.04.073 18554812

[B39] Vannucci M. Pelagatti C. Marchetti I. (2017). Manipulating cues in mind wandering: verbal cues affect the frequency and the temporal focus of mind wandering. Conscious Cogn. 53, 61–69. 10.1016/j.concog.2017.06.004 28645000

